# Notch3 Is Dispensable for Thymocyte β-Selection and Notch1-Induced T Cell Leukemogenesis

**DOI:** 10.1371/journal.pone.0024937

**Published:** 2011-09-13

**Authors:** Sara Suliman, Joanne Tan, Keli Xu, Philaretos C. Kousis, Paul E. Kowalski, Greg Chang, Sean E. Egan, Cynthia Guidos

**Affiliations:** 1 Program in Stem Cell and Developmental Biology, Hospital for Sick Children Research Institute, Toronto, Ontario, Canada; 2 Department of Immunology, Faculty of Medicine, University of Toronto, Toronto, Ontario, Canada; 3 Department of Molecular Genetics, Faculty of Medicine, University of Toronto, Toronto, Ontario, Canada; Oklahoma Medical Research Foundation, United States of America

## Abstract

Notch1 (N1) signaling induced by intrathymic Delta-like (DL) ligands is required for T cell lineage commitment as well as self-renewal during “β-selection” of TCRβ^+^ CD4*^−^*CD8*^−^* double negative 3 (DN3) T cell progenitors. However, over-expression of the *N1* intracellular domain (*ICN1*) renders N1 activation ligand-independent and drives leukemic transformation during β-selection. DN3 progenitors also express *Notch3* (*N3*) mRNA, and over-expression of ligand-independent mutant *N3* (*ICN3*) influences β-selection and drives T cell leukemogenesis. However, the importance of ligand-activated *N3* in promoting β-selection and *ICN1*-induced T cell leukemogenesis has not been examined. To address these questions we generated mice lacking functional *N3*. We confirmed that DN3 progenitors express N3 protein using a N3-specific antibody. Surprisingly however, *N3*-deficient DN3 thymocytes were not defective in generating DP thymocytes under steady state conditions or in more stringent competition assays. To determine if *N3* co-operates with *N1* to regulate β-selection, we generated *N1;N3* compound mutants. However, *N3* deficiency did not exacerbate the competitive defect of *N1^+/−^* DN3 progenitors, demonstrating that N3 does not compensate for limiting N1 during T cell development. Finally, *N3* deficiency did not attenuate T cell leukemogenesis induced by conditional expression of *ICN1* in DN3 thymocytes. Importantly, we showed that in contrast to N1, N3 has a low binding affinity for DL4, the most abundant intrathymic DL ligand. Thus, despite the profound effects of ectopic ligand-independent N3 activation on T cell development and leukemogenesis, physiologically activated *N3* is dispensable for both processes, likely because N3 interacts poorly with intrathymic DL4.

## Introduction

Notch signaling is required at multiple stages during T cell development. There are four mammalian Notch receptor paralogs (N1-4) that interact with ligands belonging to the Jagged and Delta-like families (reviewed in Ref. [1]). Ligand binding to Notch receptors induces gamma secretase-dependent cleavage within the transmembrane domain, allowing the released intracellular (ICN) domain to transit into the nucleus [2]. Nuclear ICN interacts with CSL protein bound to regulatory regions of Notch target genes, displacing transcriptional co-repressors and recruiting co-activators to induce target gene transcription. Delta-like 4 (DL4) and N1 act non-redundantly to suppress alternative hematopoietic fates of thymus-seeding progenitors [3–4–5–6–7]. N1 signaling also regulates T cell specification [8–9–10–11] as well as survival and metabolism [12] during progression to the DN3a (CD117^−^ CD25^+^ CD27^lo^ CD71^lo^) progenitor stage of intrathymic T cell development.

Development of αβ T cell progenitors beyond the DN3a stage requires successful *TCRβ* gene rearrangement and expression of the pre-TCR complex, comprised of TCRβ bound to invariant pre-Tα and CD3 proteins. Ligand-independent pre-TCR signaling initiates a developmental transition known as β-selection, in which DN3a progenitors survive, up-regulate expression of CD27, CD71 (transferrin receptor) and other receptors [13–14–15–16], and undergo blast transformation in preparation for rapid proliferation. These lymphoblasts, known as DN3b progenitors, then clonally expand and differentiate into αβ-committed CD4^+^CD8^+^ double positive (DP) thymocytes [17]–[18]. Intermediates in this transition are known as “pre-DP” thymocytes and include highly proliferative CD117^−^ CD25^−^ DN4 cells followed by CD8 immature single positive (ISP) progenitors [19].

Conditional deletion of *N1* from DN3 progenitors severely compromises generation of DP thymocytes [20], suggesting a non-redundant role for *N1* in β-selection. This role may include regulation of pre-TCR expression [20]–[21]. However, Notch signaling is also required downstream of pre-TCR expression to induce robust proliferation during the DN3-DP transition [17–22–23], likely because DL-induced Notch signaling promotes self-renewal over differentiation during the early stages of β-selection [16]. Interestingly, although *N1^+/−^* DN3 progenitors generate normal numbers of DP thymocytes at steady state, they generate very few when placed in competition with *N1^+/+^* DN3 progenitors [4]–[5]. Over-expression of Lunatic Fringe, a glycosyltransferase that enhances N1 binding to DL ligands, ameliorates this competitive defect, revealing that the size of the DP thymocyte pool is regulated by DN3 competition for limiting DL ligands in thymic niches [4–5–18]. This self-renewal role for N1 in thymofcyte β-selection likely explains why ectopic expression of ligand-independent *ICN1* in DN3 progenitors induces T cell lymphoblastic lymphoma/leukemia (T-LL) in mice [24]–[25]. Activating *N1* mutations are also very frequent in human T-LL [26], attesting to the power of *N1* as an oncogenic driver of T cell leukemogenesis.

Although N1 non-redundantly regulates T cell specification, commitment and β-selection, other Notch receptor paralogs are also expressed in T cell progenitors. Like *N1*, *N2* is required during embryogenesis [27] and is expressed in hematopoietic stem cells, DN thymocytes, and CD8 ISP cells [28]. *N2* can drive T cell development from N1-deficient hematopoietic progenitors in response to DL1 *in vitro,* but it does not do so intrathymically [29]. This failure was attributed to poor interaction of N2 with DL4, the most abundant intrathymic Notch ligand [6]–[7]. Interestingly, *N3* mRNA is sharply up-regulated at the DN3 stage of T cell development just prior to the onset of β-selection [15], suggesting it might play a role in this important developmental process. Indeed, studies of mice expressing ligand-independent mutant *ICN3* in DN3 and pre-DP thymocytes have implicated N3 in regulating pre-Tα expression and in coordinating growth and differentiation during the DN3-DP transition [30–31–32].

Notch signaling must persist beyond the DN3 stage in order to sustain self-renewing cell divisions of pre-DP thymocytes through the early phases of β-selection [16]. Thus, although N1 initiates β-selection, N3 may act downstream of N1 to regulate survival and proliferation of pre-DP thymocytes. Indeed, several examples of N1-N3 co-operation have been reported. For example *N3* deficiency in zebrafish caused defective rhombomere boundary formation in the central nervous system when *N1* was also mutant [33]. *N1* and *N3* were also reported to interact during esophageal squamous cell differentiation [34]. Importantly, *N3* has been shown to be a direct transcriptional target of *N1* in human T cell leukemia cell lines [35]–[36], revealing a potential mechanism for co-operation and suggesting that *N3* may function downstream of *N1* in T cell leukemogenesis. The ability of ectopic N3 activation in DN3 and pre-DP thymocytes to cause development of T-LL [30] is consistent with this notion.

While studies employing ectopic expression of activated N3 have been interpreted to suggest that N3 plays an important role in N1-induced thymocyte β-selection and leukemogenesis, the importance of physiologically activated N3 in these processes has not been directly evaluated. Therefore to directly assess the role of N3 in β-selection and T cell leukemogenesis, we generated *N3^LacZ/LacZ^* mice that lack functional N3 and express β-galactosidase (LacZ) from the *N3* locus. In *N3^LacZ/+^* mice, the N3/LacZ reporter was highly expressed in DN3 progenitors that co-expressed N3 protein. However, N3 protein was undetectable in *N3^LacZ/LacZ^* DN3 thymocytes, confirming that the *N3^LacZ^* allele abrogates N3 expression. Surprisingly, we detected no impact of N3 deficiency on β-selection *in vitro* or *in vivo* using stringent competition assays. Furthermore, N3 deficiency did not further reduce the competitive fitness of *N1^+/−^* DN3 progenitors, suggesting that N3 does not co-operate with N1 to regulate β-selection. Finally, *N3* deficiency did not alter the incidence or latency of T-LL induced by conditional over-expression of *ICN1* in DN3 and pre-DP thymocytes. We provide evidence that N3 has a much lower binding affinity for DL4 than N1, which suggests that N3 may not be physiologically activated by intrathymic DL4. We conclude that, despite the profound effects of ligand-independent ectopically expressed ICN3 on T cell development and leukemogenesis, physiologically activated N3 is dispensable for thymocyte β-selection and for *ICN1-*driven T cell leukemogenesis, likely because it is not efficiently activated by intrathymic DL4.

## Materials and Methods

### Mice

*N3^LacZ/LacZ^* mice were generated from embryonic stem cell line PST033 (BayGenomics, San Francisco, CA) harboring *β-geo* (fusion of β-galactosidase and neomycin phosphotransferase) inserted between exons 16 and 17 of the *N3* locus. The mice were founded on FVB background [37] and backcrossed >15 generations to C57BL/6N (CD45.2). C57BL/6N *N1^+/−^* mice have been previously described [5]. Hosts used for adoptive transfers were B6.CD45.1. Wild-type competitor donors were B6 CD45.1/CD45.2 heterozygous mice. Mice that harbor a floxed allele of *ICN1* knocked into the *Rosa26* (*R26*) locus have been previously described [38]. *R26-ICN1^lox/+^* mice were crossed to *Lck-Cre* transgenic mice as previously described [16]. All mice were bred in a specific pathogen-free facility at the Toronto Centre for Phenogenomics. Intrathymic injections were performed on anesthetized, sub-lethally irradiated (650 cGy) B6.CD45.1 mice (6–8 weeks old) as previously described [5]. All animal protocols were approved by Institutional Animal Care Committees at the Hospital for Sick Children; permit number: 6350 (Toronto, Canada), and the Toronto Centre for Phenogenomics; permit number: 12-04-0053-H (Toronto, Canada). Genotypes were determined by PCR amplification of tail DNA.

### Magnetic Bead Depletions

Thymocytes were stained with rat IgG antibodies specific for CD4 (clone YTS 191.1), CD8 (clone YTS 169.4), CD3 (clone 145-2C11), B220 (clone RA3 6B2), Mac-1 (M1/70), Gr-1 (RB6-8C5), and TER119 and cells expressing these lineage markers were depleted using goat anti-rat IgG magnetic microbeads as per the Miltenyi Biotec protocol, using an AutoMACS Pro Separator (Miltenyi Biotec, Cologne, Germany). Bone marrow (BM) cells were enriched for stem cells and lineage-negative (Lin^−^) progenitors by magnetic depletion of cells expressing CD5, CD11b, B220, 7-4, Gr-1, or TER119 using the Miltenyi Biotec Lineage Cell Depletion Kit as per the manufacturer's protocol.

### Flow Cytometry

Thymocyte single cell suspensions were made and viable cell counts determined by trypan blue exclusion. Cells were suspended in staining media and stained as previously described [5] with saturating concentrations of fluorochrome-conjugated antibodies (listed below). Fluorescence was analyzed using a BD (Becton Dickinson, San Jose, CA) LSR-II flow cytometer equipped with 4 solid-state lasers: 488nm (100 mW), 633 nm (20 mW), 405nm (25 mW) and 350 nm (20 mW). To analyze DN thymocyte subsets, Lin^−^ thymocytes were stained with saturating concentrations of flurochrome-conjugated antibodies specific for CD25 (clone 7D4) and CD117 (clone 2B8). FCS 3.0 data files were analyzed using FlowJo Versions 8.6–9.3 (Tree Star, Ashland, OR). Debris and dead cells were gated out based on low forward scatter (FSC) and high propidium iodide staining.

### Analysis of N3/LacZ Reporter Expression

β-galactosidase (LacZ) expression was detected using the Fluorescein di-β-D-galactopyranoside (FDG, Molecular Probes, Eugene, OR) substrate containing fluorescein isothiocyanate (FITC) linked to each galactose moiety, which quenches FITC fluorescence. Following enzymatic cleavage of galactose by β-galactosidase, the FITC is released from the N3/LacZ reporter and the unquenched FITC fluorescence was quantified by flow cytometry. Single cell suspensions of total or Lin^−^ DN thymocytes were hypotonically shocked to allow the entry of 2 mM FDG for 1 minute prior to addition of isotonic staining media. Cells were subsequently stained with fluorochrome-conjugated antibodies.

### Antibody Fluorochrome Conjugates

Anti-CD4-Allophycocyanin (APC, clone GK1.5), anti-CD8-eFluor650 nano-crystal (NC, clone 53–6.7), anti-CD45.1-PE-Cy7 (clone A20), and anti-CD45.2-FITC (clone 104) were purchased from eBioScience, San Diego. Anti-CD8-Phycoerythrin (PE, clone 53–6.7), anti-CD25 conjugated to FITC and APC (clone 7D4), anti-CD117-APC-Cy7 (clone 2B8), anti-CD71 conjugated to PE or biotin (clone C2), avidin-PE-Cy5, and anti-TCRβ-APC (clone H57-597) were purchased from BD Biosciences, San Jose, CA. Anti-CD4-Pacific Blue (clone RM4–5) was purchased from Invitrogen (Carlsbad, CA). Anti-mouse Notch3-PE (clone HMN3-133) and anti-human Notch3-PE (clone MHN3-21) were both purchased from BioLegend, San Diego. Polyclonal goat anti-human IgG-Fc conjugated to PE (Cedarlane, ON).

### Notch Ligand Binding Assay

To over-express N1, 293T human embryonic kidney cells were transiently transfected with pEGFP-N1-Notch1 encoding full-length murine N1 fused in-frame to GFP at the C-terminus [29]. To over-express N3, 293T cells were co-transfected with plasmids encoding human N3 with a C-terminal Myc tag (pCDNA4-His-Myc-A-hNotch3) and pmaxFP-Green-N (pMAX-GFP) from Amaxa-Lonza (Walkersville, MD). Transfections were performed using FuGene (Roche, Mississauga) according to the manufacturer's protocol. After 72 hrs, flow cytometry was used to assess transfectants for binding of control hFc versus DL4-Fc (kindly provided by Dr. Freddy Radtke) fusion proteins as previously described [29]. Surface expression of N3 on GFP versus N3-Myc+GFP transfectants was evaluated by flow cytometry using an anti-human N3 Ab as described above. In addition, whole cell lysates from transfected and control untransfected 293T cells were prepared using RIPA buffer (50mM Tris-HCl, 1% NP-40, 0.25% Sodium deoxycholate, 150mM NaCl, 1mM EDTA pH 7.4) and protease inhibitors (1mM sodium vanadate and 1mM sodium fluoride). 10 µg of each lysate was used for immunoblotting with anti-human c-Myc monoclonal antibody clone 9E10 (Cedarlane Laboratories Ltd, Burlington, ON) and rabbit polyclonal anti-β-actin antibody, was purchased from Sigma-Aldrich (St. Louis, MO).

### *In Vitro* T Cell Differentiation

DN3 (Lin^−^ CD117^−^ CD25^+^) progenitors from each genotype were sorted directly into 24-well plates (2×10^3^/well) containing OP9-DL4 stromal cells [16] (2×10^4^/well plated the previous day) and cultured for 3 or 6 days as previously described [16]. After each time point viable cell counts were determined by trypan blue exclusion and expression of CD4 and CD8 was assessed by flow cytometry.

### Characterization of T-Lymphoblastic Leukemia (T-LL)

Cohorts of *N3^+/+^ R26-ICN1^lox/+^;Lck-Cre^+^ vs N3^LacZ/LacZ^ R26-ICN1^lox/+^;Lck-Cre^+^* mice were aged until they became moribund and showed clinical signs of leukemia, such as scruffiness, distended abdomen, and/or lethargy. Livers, kidneys and spleens were fixed in 4% formaldehyde and processed for hematoxylin and eosin staining using standard procedures. Thymi, spleens, lymph nodes and bone marrow (BM) of moribund mice were explanted and analyzed for expression of GFP and TCRβ using flow cytometry as described above. To determine if the T cell leukemias were transplantable, 3X10^6^ GFP^+^ cells were sorted from spleen and lymph nodes of moribund mice and injected intravenously into sub-lethally irradiated (650 cGy) *RAG2^−/−^*; *Ly5.2* hosts. Leukemic burden was assessed 2 and 4 weeks later using flow cytometry to evaluate the abundance of GFP^+^ TCRβ leukemic blasts in spleen, lymph nodes and bone marrow of injected mice.

### Statistical Methods

For all experiments, two-tailed unpaired Student *T-*test with Welch's correction (assuming unequal variances) was used with a 95% confidence interval for pair-wise comparisons between different genotypes. For Kaplan-Meier survival curves, Student *T-*test used to compare the difference in the survival between the two groups. The median survival of each genotype was also calculated. All statistical analyses were performed using Prism software (V4.0).

## Results

### DN3 thymocytes from *N3^LacZ/LacZ^* mice lack N3 expression

**Figure 1:**
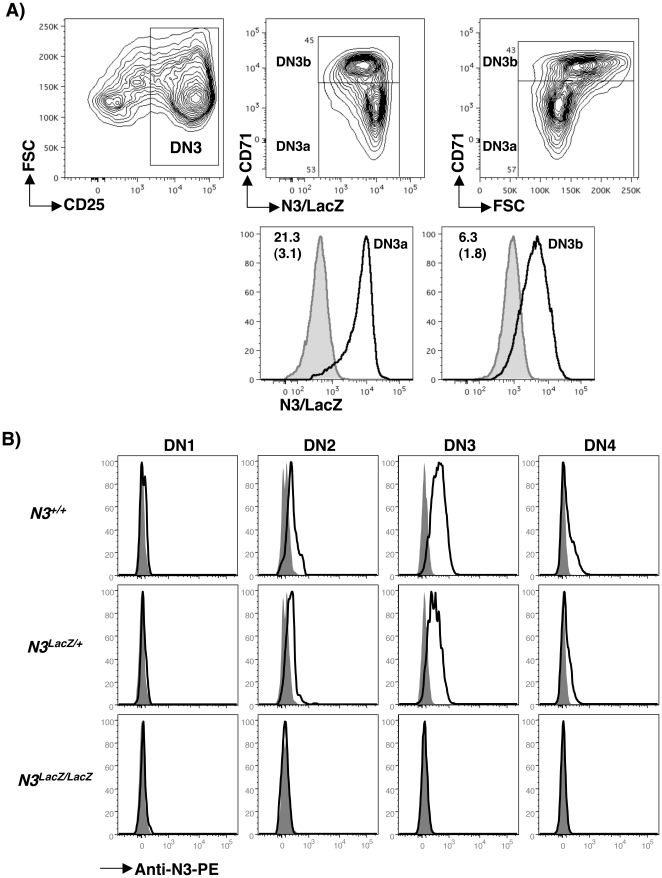
N3/LacZ reporter expression in DN3 thymocytes. (A) Lin- DN thymocytes from N3LacZ/LacZ and N3+/+ mice were loaded with FDG and stained with anti-CD25-APC, anti-CD71-biotin, and anti-CD117-APCCy7 followed by Avidin-PECy5. Top: Left plot shows FSC vs CD25 distribution of CD117- DN thymocytes from N3LacZ/LacZ mice. CD25+ DN3 thymocytes (rectangular gate) were gated to display N3/LacZ vs CD71 (middle) and FSC versus CD71 (right). Rectangles in the middle and right plots show gates used to define DN3a (CD71lo), and DN3b (CD71hi) subsets. Bottom: Histograms show N3/LacZ reporter expression in DN3a and DN3b thymocytes from N3LacZ/LacZ mice (open histograms) compared to background FITC autofluorescence in each N3+/+ subset (shaded histograms). Numbers in each plot depict the average ratio of FITC median fluorescence intensity from N3LacZ/LacZ mice divided by FITC median intensity of the same subset from N3+/+ mice. Numbers in brackets are the standard deviation of this measurement (N = 3 per genotype). (B) Surface expression of N3 protein on DN thymocytes. Open histograms depict anti-N3-PE staining on DN1 (CD117^+^ CD25^−^), DN2 (CD117^+^ CD25^+^), DN3 (CD117^−^ CD25^+^), and DN4 (CD117^−^ CD25^−^) thymocytes from *N3^+/+^* (top), *N3^LacZ/+^* (middle) and *N3^LacZ/LacZ^* (bottom) mice compared to “fluorescence minus one” controls generated by staining *N3^+/+^* thymocytes with all antibodies except anti-N3-PE (shaded histograms). These experiments were repeated twice showing similar results.

To study N3 expression and function in T cell development, we generated mice from embryonic stems cells in which *N3* is fused in frame with β-galactosidase (LacZ), generating a null allele of *N3* and a reporter for *N3* gene expression [37]–[39]. Upon challenge, *N3^LacZ/LacZ^* mice show increased susceptibility to ischemic stroke [40], but when unperturbed they appear normal and healthy, allowing us to determine how loss of *N3* function impacts T cell development. We employed a sensitive fluorogenic substrate to detect N3/LacZ reporter expression in progenitors just before and after the β-selection checkpoint. Interestingly, N3/LacZ expression was higher in DN3a (CD71^lo^) than in CD71^hi^ DN3b thymocytes from *N3^LacZ/+^* mice (Fig. 1A, top), in keeping with a similar decline in *N3* mRNA during this transition [15]–[41]. However, background levels of auto-fluorescence were higher in DN3b thymocytes (Fig. 1A, bottom). Therefore, in order to directly compare changes in N3/LacZ reporter expression between subsets, we normalized fluorescence intensities of *N3^LacZ/+^* cells to the background fluorescence of *N3^+/+^* cells for each subset. Comparing these normalized ratios revealed that on average, N3/LacZ expression was highest in DN3a cells and declined 3-4-fold in DN3b thymocytes.

Importantly, these subtle changes in N3/LacZ reporter expression are highly correlated with previously reported changes in *N3* mRNA expression across this transition [15]–[41], revealing that N3/LacZ “reports” *N3* expression accurately. Furthermore, N3 protein was most highly expressed on DN2 and DN3 thymocytes from *N3^+/+^* mice, but declined dramatically by the DN4 stage (Fig. 1B) and was not detected on DP thymocytes (data not shown). Thus, similar to *N3* mRNA [15]–[41], expression of N3 protein is sharply induced just prior to the β-selection checkpoint but declines precipitously thereafter.

Prior studies have demonstrated that the *N3^LacZ^* allele does not produce detectable levels of mRNA or protein [39–40–42]. Furthermore, *N3^LacZ/LacZ^* mice exhibit vascular smooth muscle defects [40], confirming loss of N3 function in these mice. We used a N3-specific antibody to examine N3 protein expression in thymocytes from *N3^LacZ/LacZ^* mice. N3 protein expression was slightly lower in all subsets from *N3^LacZ/+^* mice indicating a gene dosage effect (Fig. 1B). Importantly, we could not detect N3 protein expression on any thymocyte subsets from *N3^LacZ/LacZ^* mice (Fig. 1B and data not shown). Thus, N3 protein is highly expressed in DN3 thymocytes from *N3^+/+^* and *N3^LacZ/+^* but not *N3^LacZ/LacZ^* mice.

### N3 is dispensable for thymic development at steady state and in competition with *N3^+/+^* DN3 progenitors

**Figure 2:**
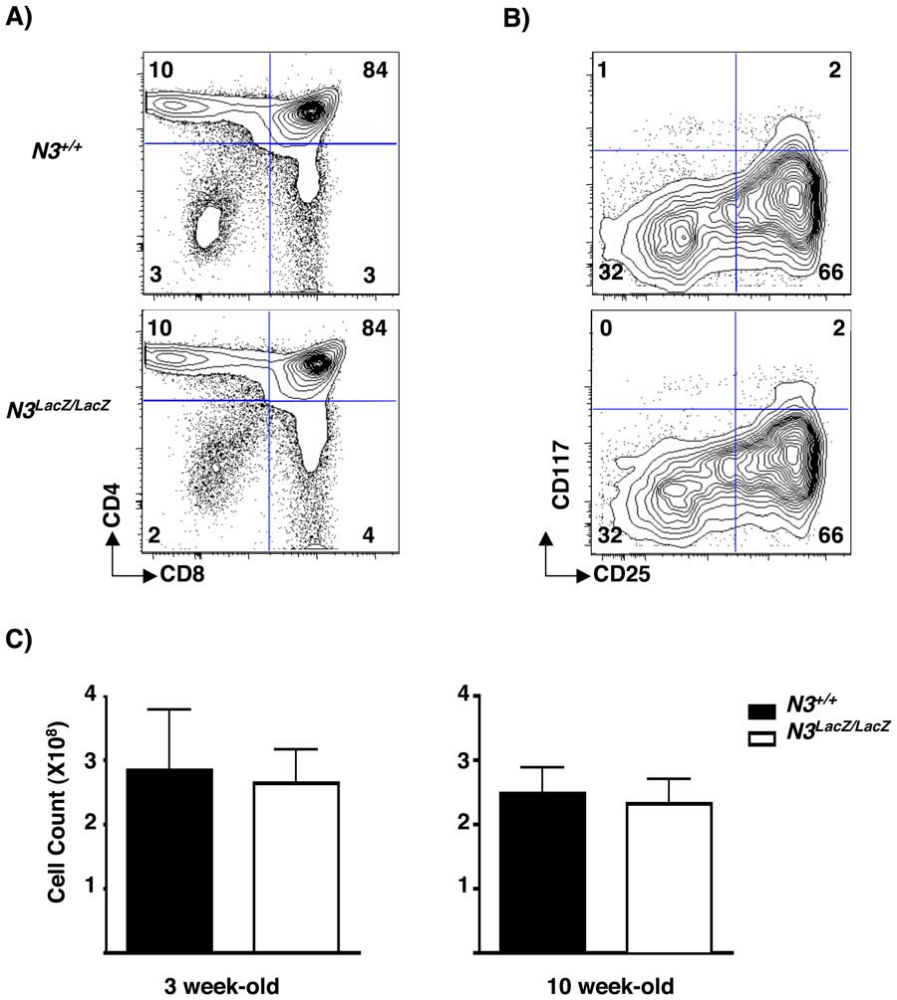
Steady-state thymic immunophenotype and cellularity in *N3^LacZ/LacZ^* mice. (A) CD4 vs CD8 distribution of total thymocytes from *N3^+/+^* and *N3^LacZ/LacZ^* mice. Thymocytes were stained with anti-CD4-APC and anti-CD8-PE antibodies and analyzed by flow cytometry. (B) CD117 vs CD25 distribution of Lin^-^ DN thymocytes from *N3^+/+^* and *N3^LacZ/LacZ^* mice. (C) Total thymic cellularity of *N3^+/+^* (black bars) vs *N3^LacZ/LacZ^* (white bars) of 3 week-old (left) and 10 week-old mice (right). Two-tailed Student *T*-test analyses: *P* = 1 for 3 week-old *N3^+/+^* (N = 4) vs *N3^LacZ/LacZ^* (N = 4) mice, and *P* = 0.8 for 10 week-old *N3^+/+^* (N = 4) vs *N3^LacZ/LacZ^* (N = 5) mice.

To determine if N3 deficiency impacts β-selection, we analyzed the relative and absolute numbers of intrathymic T cell progenitors in *N3^+/+^* and *N3^LacZ/LacZ^* littermates. Although another study of 129/Sv *N3*-deficient mice reported a minor reduction in thymic cellularity at 10-weeks of age [43], we did not observe lower percentages or absolute numbers of DN, or post-β-selection subsets at steady state in C57BL6 *N3^LacZ/LacZ^* relative to *N3^+/+^* mice at either 3 or 10 weeks of age (Fig. 2).

**Figure 3:**
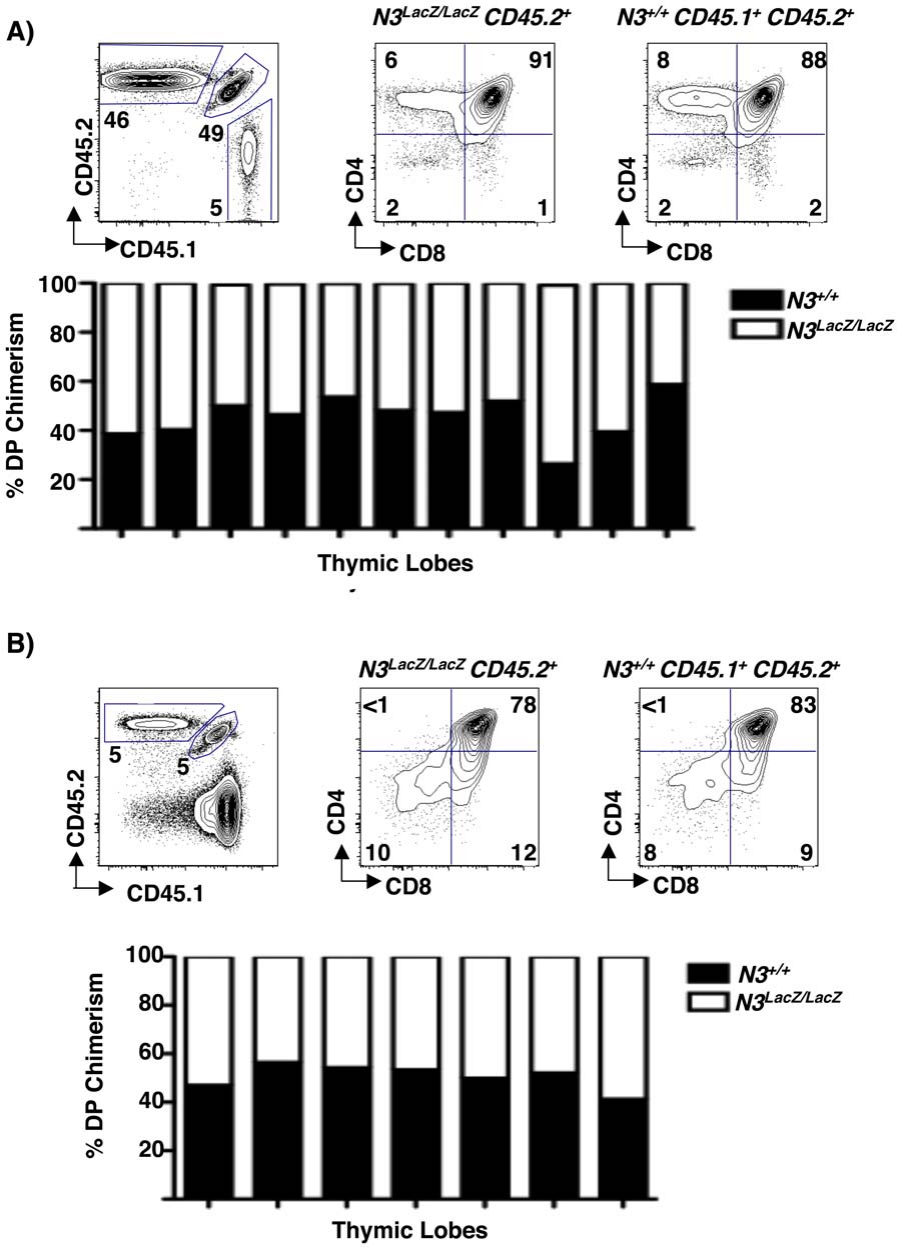
Normal competitive fitness of *N3^LacZ/LacZ^*T cell progenitors. (A) Equal mixtures of Lin^-^ BM progenitors from *N3^+/+^* and *N3^LacZ/LacZ^* donors were co-injected intrathymically into sub-lethally irradiated *B6.CD45.1* hosts (10^5^ cells/thymic lobe). Three weeks later, thymocytes were stained with anti-CD45.1-PECy7, anti-CD45.2-FITC, anti-CD4-APC and anti CD8-PE and analyzed by flow cytometry. Top: Representative example showing CD45.1 vs CD45.2 distribution of total thymocytes and CD4 vs CD8 distributions of CD45.1^+^ CD45.*2^+^* (*N3^+/+^*) and CD45.2^+^ (*N3^LacZ/LacZ^*) donor-derived thymocytes. Bottom: Relative contribution of *N3^+/+^* (black bars) vs *N3^LacZ/LacZ^* (white bars) to the DP thymocyte population in individual thymic lobes. Two-tailed Student *T*-test of the relative contribution of *N3^+/+^* vs *N3^LacZ/LacZ^* donors showed no significant difference (*P* = 0.95). (B) Equal mixtures of DN3 progenitors sorted from *N3^+/+^* and *N3^LacZ/LacZ^* mice were co-injected intrathymically into sub-lethally irradiated B6.CD45.1 hosts (10^5^ cells/lobe). One week later, thymocytes were stained and analyzed by flow cytometry as described for Figure 3A. Top: Representative example showing CD45.1 vs CD45.2 distribution of total thymocytes and CD4 vs CD8 distributions of *N3^+/+^* and *N3^LacZ/LacZ^* donor-derived thymocytes. Bottom: Bar graph depicts relative contribution of *N3^+/+^* (black bars) vs *N3^LacZ/LacZ^* (white bars) to the DP thymocyte population of individual thymic lobes. Two-tailed Student *T*-test of the relative contribution of *N3^+/+^* vs *N3^LacZ/LacZ^* donors showed no significant difference (*P* = 0.94). This experiment was repeated twice with similar results.

Competition amongst DN3 progenitors for DL Notch ligands in thymic niches regulates the size of the DP thymocyte pool [4–5–18]. We next examined DP thymocyte production from *N3^LacZ/LacZ^* DN3 progenitors under more stringent competitive conditions, which can reveal developmental defects not evident at steady state [5]–[44]. We therefore reasoned that *N3* deficiency might compromise the ability of DN3 progenitors to compete for Notch-ligand bearing thymic niches even though N3 was dispensable for steady state thymopoiesis. To test this idea, we injected mixtures containing equal numbers of *N3^+/+^ CD45.1; CD45.2* and *N3^LacZ/LacZ^ CD45.2* Lin^-^ BM progenitors into individual thymic lobes of sub-lethally irradiated wild-type B6 CD45.1 hosts. The contribution of each donor population to the DP thymocyte pool was assessed 3 weeks later. Thymocytes derived from both donor genotypes were highly abundant and most had progressed to the DP and mature CD4 single positive stages (Fig. 3A, top). However, on average, *N3^LacZ/LacZ^* and wild-type donors contributed equally to the DP (Fig. 3A, bottom) and mature CD4^+^ and CD8^+^ thymocyte pools (data not shown).

We considered the possibility that robust proliferation of DN1 and DN2 progenitors derived from Lin^−^ BM donors might generate enough DN3 progenitors to saturate Notch ligand-bearing niches before N3 expression was fully induced. Therefore we repeated the competitive repopulation assay using DN3 progenitors purified from *N3^LacZ/LacZ^ CD45.2* and *N3^+/+^ CD45.1; CD45.2* donors. Equal mixtures of the two donor DN3 populations were intrathymically injected into sub-lethally irradiated B6.CD45.1 hosts. Because we started with more developmentally advanced progenitors, the injected mice were analyzed after 1 week, a time-point previously shown to yield maximal repopulation from DN3 progenitors [45]. Once again, both genotypes contributed equally to the donor-derived DP thymocyte pool (Fig. 3B). Thus, N3 deficiency does not compromise the ability of DN3 progenitors to access Notch ligand-bearing intrathymic niches under competitive conditions.

### No evidence for N3 co-operation with N1 during β-selection

**Figure 4:**
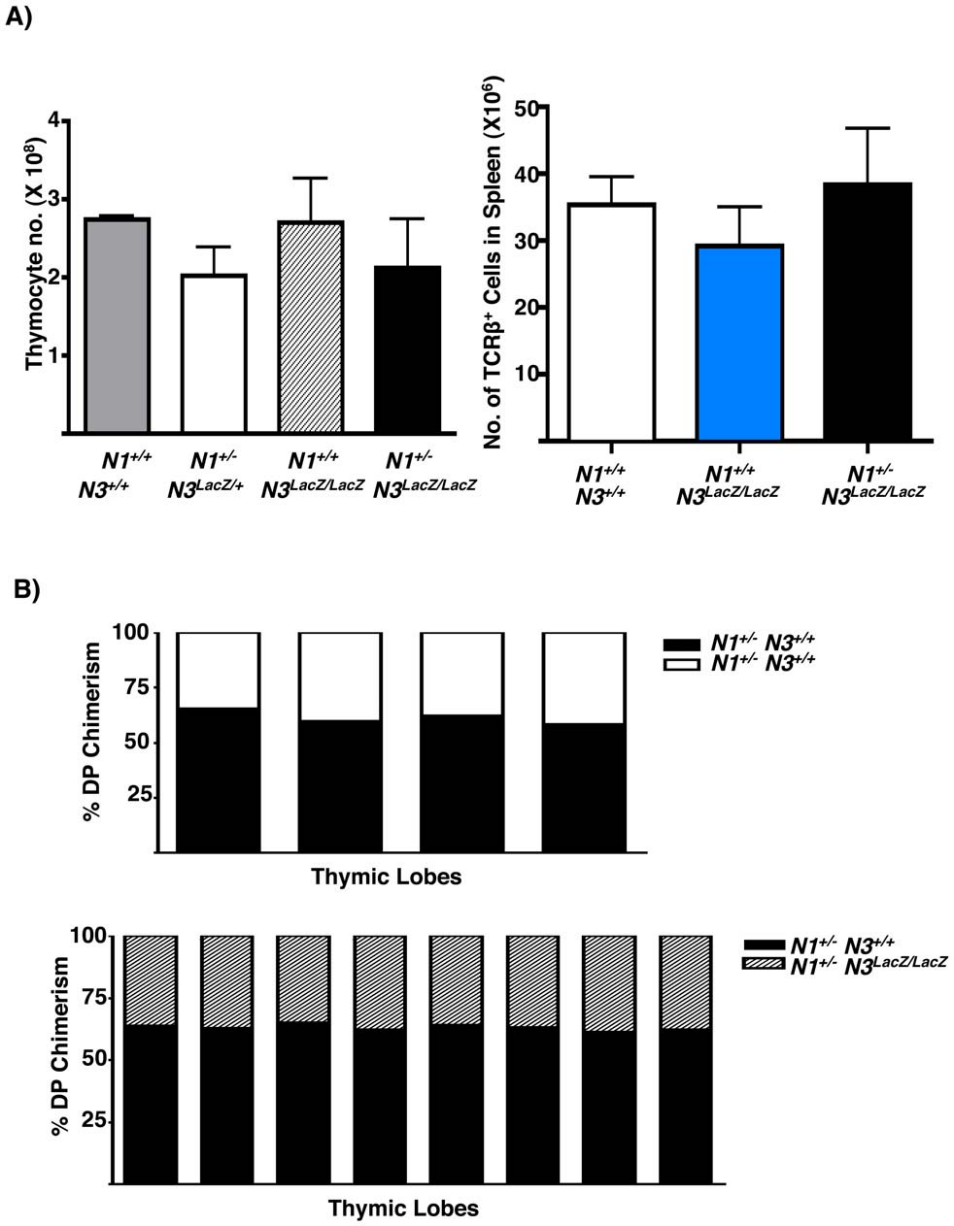
No Evidence for N1-N3 Cooperation during β-selection. (A) Effect of reduced N1 gene dosage on steady state thymocyte and splenic T cell numbers in *N3^LacZ/LacZ^* mice: Left bar graph shows mean total thymic cell counts from *N1^+/+^ N3^+/+^* (grey, N = 3), *N1^+/−^ N3^LacZ/+^* (white, N = 4), *N1^+/+^ N3^LacZ/LacZ^* (hatched, N = 4), and *N1^+/−^ N3^LacZ/LacZ^* (black, N = 6) mice at 6 weeks of age. Two-tailed Student's *T*-test comparisons gave *P* values ranging from 0.2–1 for all pair-wise comparisons. Right bar graph shows average number of TCRβ^+^ splenic T cells at 6–8 weeks of age in *N1^+/+^ N3^+/+^* (white, N = 3), *N1^+/+^ N3^LacZ/LacZ^* (blue, N = 4), and *N1^+/−^ N3^LacZ/LacZ^* (black, N = 6) mice. Two-tailed Student's *T*-test comparisons: *N1^+/+^ N3^+/+^* vs *N1^+/+^ N3^LacZ/LacZ^ (P* = 0.6), *N1^+/+^ N3^LacZ/LacZ^* vs *N1^+/−^ N3^LacZ/LacZ^* (*P = *0.4), and *N1^+/+^ N3^LacZ/LacZ^* vs *N1^+/−^ N3^LacZ/LacZ^* (*P = *0.6). (B) N3 does not compensate for lower *N1* gene dose in *N1^+/−^* thymocytes. Top: Equal mixtures of *N1^+/−^ N3^+/+^ CD45.1;CD45.2* (black bars) and *N1^+/−^ N3^+/+^ CD45.2* (white bars) DN3 progenitors co-injected intrathymically into sub-lethally irradiated B6.CD45.1 hosts. One week later, cells from each thymic lobe were stained with anti-CD45.1-PECy7, anti-CD45.2-FITC, anti-CD4-Pacific Blue and anti CD8-PE and analyzed by flow cytometry. The average ratio of CD45.1^+^ CD45.2^+^ to CD45.2^+^ DP progeny was 1.6±0.2. Bottom: Equal mixtures of *N1^+/−^ N3^+/+^ CD45.1;CD45.2* (black bars) and *N1^+/−^ N3^LacZ/LacZ^ CD45.2* (dashed bars) DN3 progenitors were co-injected intrathymically into sub-lethally irradiated B6.CD45.1 host. One week later, flow cytometry analysis of each lobe shows that the average ratio of CD45.1^+^ CD45.2^+^ to CD45.2^+^ DP progeny was 1.5±0.2. Two-tailed Student *T*-test comparison of the ratios from each type of competition: (*P* = 0.5). The experiment was performed three times yielding similar results.

The normal generation of DP thymocytes from of *N3^LacZ/LacZ^* DN3 progenitors under steady state and competitive conditions may reflect potent compensation by N1. Similar N1 compensation for N4 deficiency has been reported [46]. To formally evaluate functional redundancy or cooperation between N1 and N3, we intercrossed *N1^+/−^* and *N3^LacZ/LacZ^* mice to generate compound mutants. We hypothesized that if N1 and N3 play partially redundant or co-operative roles in T cell development, then *N1^+/−^ N3^LacZ/LacZ^* mice should show a more severe steady state defect in thymic lymphopoiesis than either *N1^+/−^ N3^LacZ/+^* or *N1^+/+^ N3^LacZ/LacZ^* mutants. However, at steady state there was no significant difference in thymus cellularity or splenic T cell abundance in *N1^+/−^ N3^LacZ/LacZ^* mice relative to either *N1^+/−^ N3^LacZ/+^* or *N1^+/+^ N3^LacZ/LacZ^* littermate controls (Fig. 4A).

Previous studies have shown that *N1^+/−^* T cell progenitors show compromised production of DP thymocytes when placed in competition with wild-type *N1^+/+^* progenitors [5]–[11]. We reasoned that a role for *N3* in β-selection might be more obvious when N1 gene dose is limiting. To test this notion we generated compound *N1^+/−^ N3^LacZ/LacZ^* mutants, and carried out competition experiments with equal mixtures of *N1^+/−^ N3^LacZ/LacZ^ CD45.2* and *N1^+/−^ N3^+/+^ (CD45.1; CD45.2)* DN3 progenitors. Thus in this scenario, both donor subsets were *N1* heterozygous but they differed in *N3* gene dose. For this experiment, control competitions were performed with equal mixtures of *N1^+/−^ N3^+/+^ (CD45.2)* plus *N1^+/−^N3^+/+^(CD45.1; CD45.2)* DN3 cells. The average ratio of DP progeny derived from the 2 donors was 1.6±0.2 in the control competitions (Fig. 4B, top). Importantly, this ratio was not significantly different (p = 0.5) from the ratio of 1.5±0.2 derived from competitions between *N1^+/−^ N3^LacZ/LacZ^* and *N1^+/−^ N3^+/+^* donors (Fig. 4B, bottom). These data demonstrate that *N3* does not compensate for limiting *N1* gene dose during β-selection, even under stringent competitive conditions.

### N3 is dispensable for *ICN1*-induced T cell leukemogenesis

**Figure 5:**
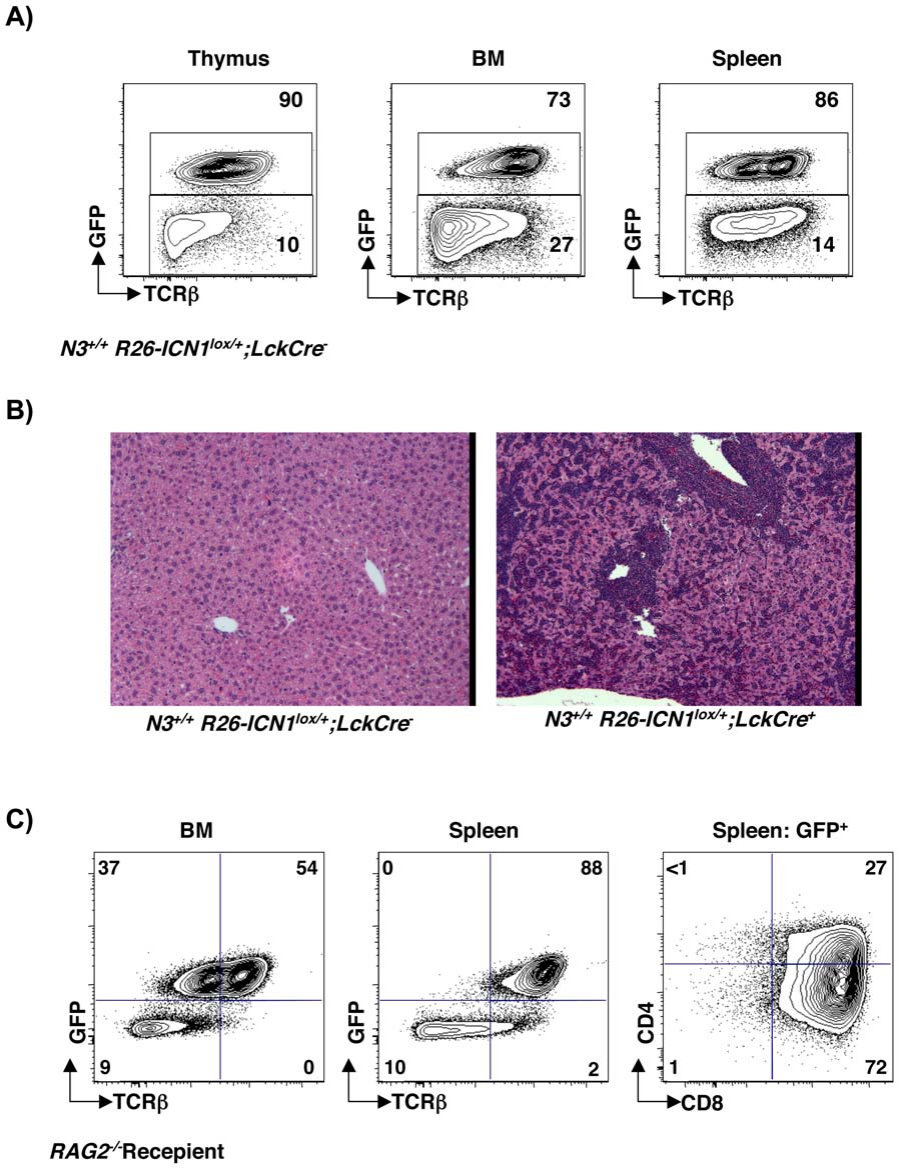
*N3^+/+^ Lck-Cre^+^ R26-ICN1 ^lox/+^* mice develop transplantable T-LL. (A) Infiltration of lymphoid organs in moribund *N3^+/+^ Lck-Cre^+^ R26-ICN1^lox/+^* mouse by eGFP^+^ TCRβ^+^ lymphoblasts. Cells were isolated from various lymphoid organs and stained with anti-TCRβ-PECy7 antibody. Plots depict eGFP vs. TCRβ-PECy7 in thymus (left), bone marrow (middle) and spleen (right). (B) Hematoxylin and eosin stained liver sections from *N3^+/+^;Lck-Cre^−^*; R*26-ICN1^lox/+^* (left) and moribund *N3^+/+^; Lck-Cre^+^*; *R26-ICN1^lox/+^* (right) mice showing dark aggregates of leukemic lymphoblasts around hepatic vessels. (C) eGFP^+^ TCRβ^+^ lymphoblasts were sorted from spleens and lymph nodes of moribund *N3^+/+^ Lck-Cre^+^ R26-ICN1^+^* mice and intravenously injected into sub-lethally irradiated *RAG2^−/−^* mice. After two weeks, cells from the indicated organs were isolated and stained with anti-TCRβ-PECy7, anti-CD4-Pacific Blue and anti-CD8-eFluor650 antibodies. TCRβ vs. eGFP plot shows infiltration of bone marrow (left) and spleen (middle) with eGFP^+^ cells in host *RAG2^−/−^* recipient mice. Plot on the left shows CD8 vs. CD4 distribution gated on eGFP^+^ cells in the spleen.

Previous studies have shown that transgenic mice expressing ligand-independent mutant *ICN3* in DN3 and pre-DP thymocytes under control of the *Lck* proximal promoter develop T-LL [30]–[31]. In addition, *N3* expression is directly regulated by ICN1 in human T-LL cells [35]–[36]. To determine if *N3* is required for ICN1-induced T cell leukemogenesis, we used mice that harbor a ligand-independent constitutively active *ICN1* allele knocked into the *R26* locus [38]. This *ICN1* allele is preceded by a lox-stop-lox sequence and is followed by an *IRES* and *eGFP* cDNA. Based on studies of GFP expression in Cre reporter mice, *Lck-Cre* activity is robust in DN3 thymocytes but not prior to that developmental stage [47]. As expected [24]–[25], targeting *ICN1* to DN3 thymocytes caused *R26-ICN1^lox/+^;Lck-Cre^+^* mice to become moribund due to development of T-LL. Clinical signs of T-LL included splenomegaly and enlarged lymph nodes. Lymphoid tissues and BM were highly infiltrated with eGFP^+^ TCRβ^+^ lymphoblasts (Fig. 5A). Histological analyses also revealed extensive infiltration of liver by leukemic lymphoblasts (Fig. 5B). Importantly, eGFP^+^ TCRβ^+^ could adoptively transfer T-LL to immune-deficient *RAG2^−/−^* mice (Fig. 5C).

**Figure 6:**
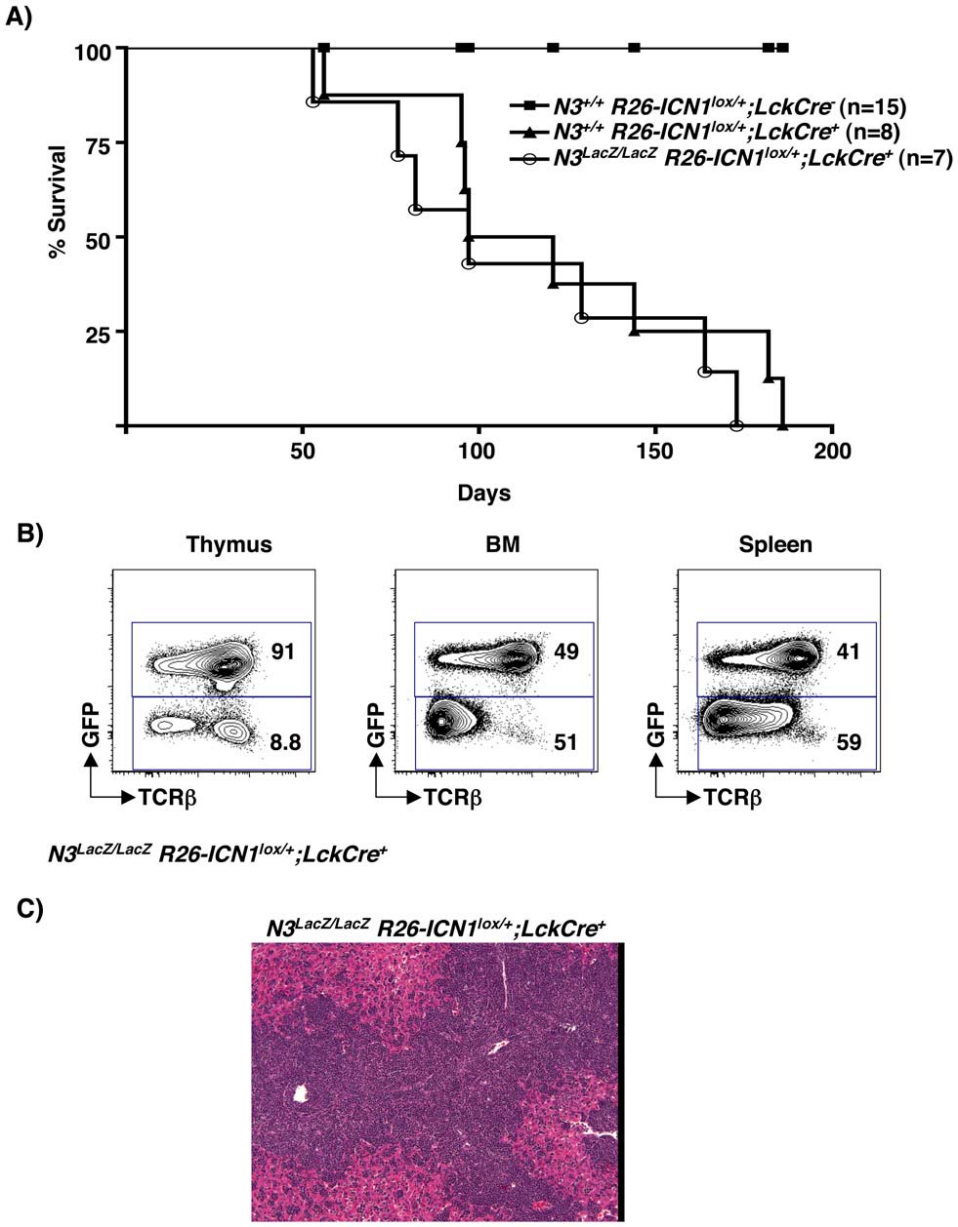
N3 is dispensable for *ICN1-*induced T cell leukemogenesis. (A) Kaplan-Meier survival curve for *N3^+/+^ R26-ICN1^lox/+^;Lck-Cre^−^* (closed squares), *N3^+/+^ R26-ICN1^lox/+^;Lck-Cre^+^* (closed triangles), and *N3^LacZ/LacZ^ R26-ICN1^lox/+^;Lck-Cre^+^* (open circles) mice over 200 days. Two-tailed Student *T-*test comparison between the latter two strains showed no statistically significant difference (P = 0.4). (B) Lymphoid organ infiltration by leukemic T cell blasts in a moribund *N3^LacZ/LacZ^ R26-ICN1^lox/+^;Lck-Cre^+^* mouse, analyzed as described in Fig. 5. (C) Hematoxylin and eosin stained liver section from *N3^LacZ/LacZ^ R26-ICN1^lox/+^;Lck-Cre^+^* mouse shows dark aggregates of leukemic lymphoblasts around hepatic vessels.

To determine if *N3* contributes to *ICN1*-induced T-LL in this model, we evaluated T-LL induction in *N3^+/+^ R26-ICN1^lox/+^;Lck-Cre^+^ vs N3^LacZ/LacZ^ R26-ICN1^lox/+^;Lck-Cre^+^* mice. *N3^+/+^ R26-ICN1^lox/+^;Lck-Cre^+^* mice developed T-LL with a median latency of 109±16.1 days (Fig. 6A). Importantly, N3-deficiency did not impair this process, since *N3*-deficient *R26-ICN1^lox/+^;Lck-Cre^+^* mice developed clinical signs of T-LL with a similar median latency of 97±17.3 days (*P* = 0.4). Furthermore, clinical features of T-LL lacking *N3* were indistinguishable from those seen in *N3^+/+^ R26-ICN1^lox/+^;Lck-Cre^+^* mice (Fig. 6B, C). Based on these findings, we conclude that *N3* is dispensable for *N1*-induced leukemogenesis.

### Differential binding of DL4 by N1 and N3

**Figure 7:**
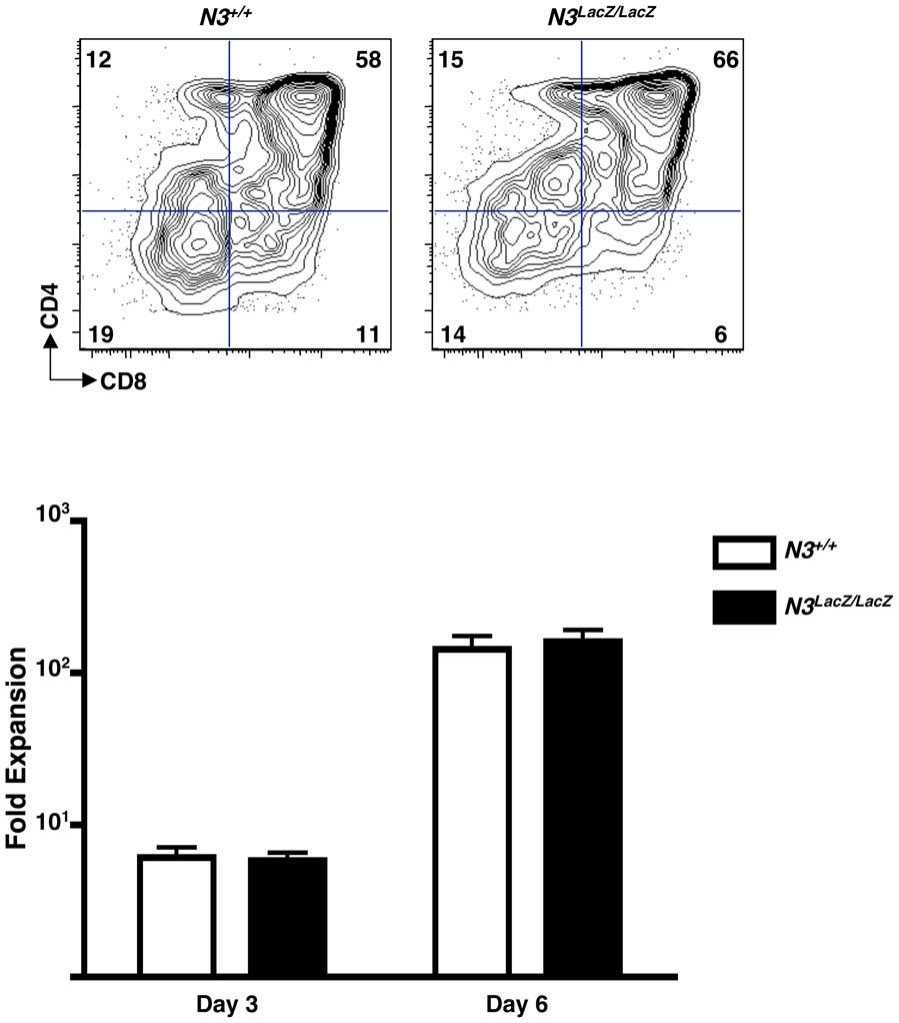
Effect of *N3* deficiency on DL4-induced β-selection *in vitro*. *N3^LacZ/LacZ^* DN3 thymocytes show normal DL4-induced proliferation and differentiation *in vitro.* DN3 thymocytes from either *N3^+/+^* or *N3^LacZ/LacZ^* mice were co-cultured with OP9-DL4 stromal cells for 3 or 6 days. At each time point, viable cell counts were determined and cultures were stained with anti-CD4-APC and anti-CD8-PE antibodies. Top: Representative CD4 vs CD8 plots from 6-day progeny of *N3^+/+^* (left) and *N3^LacZ/LacZ^* (right) DN3 thymocytes. Bottom: Fold-expansion of *N3^+/+^* (white bars) and *N3^LacZ/LacZ^* (black bars) DN3 thymocytes co-cultured with OP9-DL4 on day 3 (left, *P* = 0.8*)* and day 6 (right, *P* = 0.7*).*

DL4 acts non-redundantly to promote T lineage specification at early stages of T cell development [6]–[7]. Although *N2* is expressed in early T cell progenitors, it cannot compensate for the developmental defects caused by *N1* deficiency [29]. The ability of N1 but not N2 to promote T cell development *in vivo* has been attributed to the more robust avidity of N1 for DL4, as assessed by measuring binding of DL4-Fc fusion protein to transiently transfected N1 vs N2 [29]. We speculated that the failure of *N3* deficiency to impact thymocyte β-selection or ICN1-induced T cell leukemogenesis might indicate that N3 is not efficiently activated by DL4. Consistent with this notion, we found that *N3* deficiency did not impair the ability of DN3 progenitors to proliferate and differentiate into DP thymocytes after co-culture with OP9-DL4 stromal cells *in vitro* (Fig. 7). This finding suggests that N3 does not significantly contribute to DL4-induced β-selection in DN3 and pre-DP progenitors.

**Figure 8:**
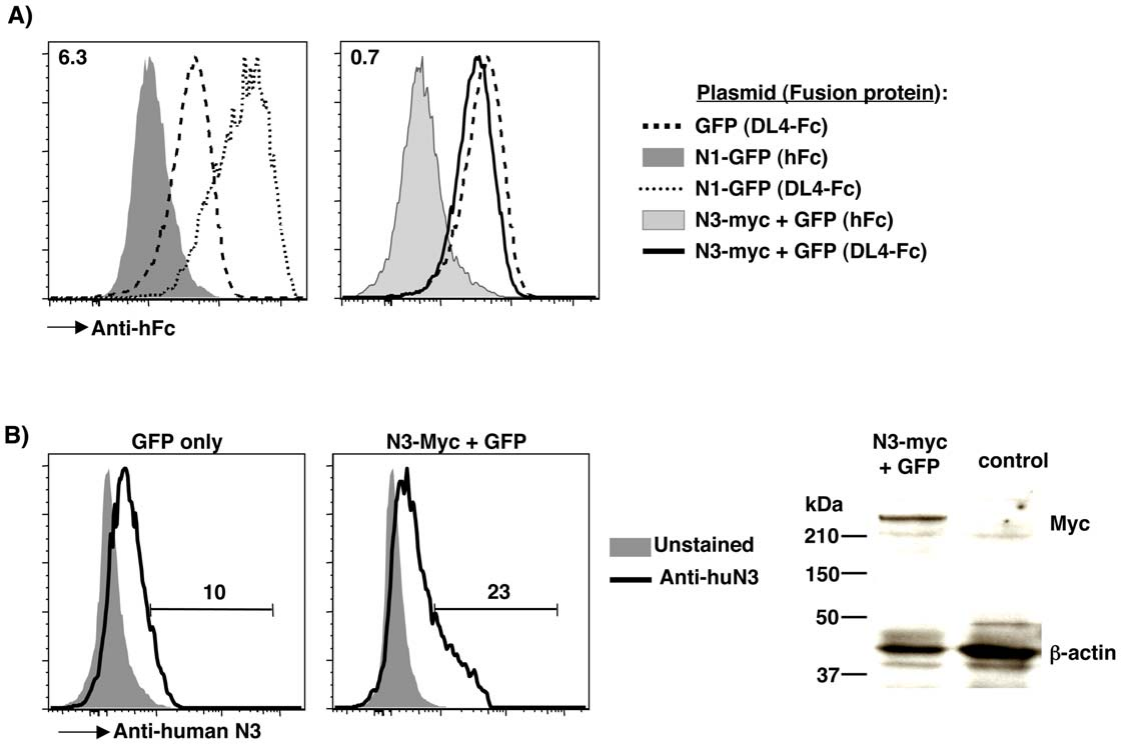
Comparison of DL4 binding to N1 versus N3. 293T cells were transiently transfected with plasmids encoding GFP, N1-GFP or N3-Myc+GFP. (A) After 72 hrs, transfectants were incubated with control hFc vs DL4-Fc fusion proteins followed by PE-conjugated anti-human Fc antibody and evaluated by flow cytometry. Histograms show PE fluorescence of live single GFP^+^ cells. Left: DL4-Fc binding to 293T cells transfected with GFP alone (dashed line) versus N1-GFP transfectants (dotted line). Control hFc binding to N1-GFP transfectants (dark grey, filled) is also shown for comparison. Right: DL4-Fc binding to GFP (dashed line) versus N3-Myc+GFP transfectants (solid line). Control hFc binding to N3-Myc+GFP transfectants (light grey, filled) is also shown. The median fluorescence intensity ratio of DL4-Fc staining for N1-GFP/GFP DL4-Fc (left) and N3-Myc+GFP/GFP (right) transfectants are shown in the upper left of each plot. (B) Validation of N3-Myc expression in N3-Myc+GFP 293T transfectants. 293T cells transfected with the indicated plasmids were evaluated after 72 hrs for human N3 expression by flow cytometry and immunoblotting. Histograms show PE fluorescence from GFP transfectants (left) or N3-Myc+GFP transfectants (middle) unstained (dark grey, filled) versus stained with PE-conjugated anti-human N3 antibody (black line). All histograms were pre-gated to show live single GFP^+^ cells. Right: Lysates from the indicated transfectants were immunoblotted with anti-human c-Myc (top) and β-actin (bottom) antibodies. The expected molecular weight of Myc-tagged human N3 is 230 kDa versus 42 kDa for β-actin.

We hypothesized that the failure of *N3* deficiency to impact DL4-induced β-selection might reflect poor binding of DL4 to N3. To test this possibility, we compared the ability of N1 and N3 to bind DL4-Fc fusion protein using a transient transfection assay [29]. 293T cells showed slightly higher binding of DL4-Fc than the control Fc protein, indicating expression of endogenous Notch receptors (Fig. 8A, left). However, we observed 6-fold greater binding of DL4-Fc to N1-GFP transfected cells relative to GFP transfected cells, indicating that N1 over-expression greatly enhances DL4-Fc binding to 293T cells, as expected based on a previous study [29]. In contrast, N3-Myc + GFP transfected cells showed similar levels of DL4-Fc binding to control GFP-transfected 293T cells (Fig. 8A, right). We validated expression of transfected human N3 protein by surface staining and immunoblotting (Fig. 8B). Therefore, these data suggest that N3 has a much lower binding affinity for DL4 than N1. In light of these findings, we suggest that the failure of *N3* to compensate for reduced *N1* gene dosage during thymocyte β-selection reflects inefficient N3 activation by intrathymic DL4.

#### Discussion

The aim of this study was to determine if N3 signaling is required during thymocyte β-selection or during *N1*-induced T cell leukemogenesis. We utilized *N3/LacZ* “knock-in” reporter mice that lack detectable *N3* mRNA [39] and showed that they lack N3 protein. The N3/LacZ reporter as well as N3 protein was highly expressed in *N3^+/+^* DN3 progenitors, in agreement with studies on *N3* mRNA expression [15]–[41]. Nonetheless, we observed no impact of *N3* deficiency on thymocyte β-selection at steady state or under more stringent competitive repopulation experiments. In contrast, DN3 competitive fitness was severely impaired by loss of only one *N1* allele. However, *N3* deficiency did not further decrease the ability of *N1^+/−^* DN3 progenitors to compete for Notch ligand niches in the thymus. Thus, although *N3* is highly expressed in DN3 thymocytes, its loss had no impact on β-selection, even when *N1* was limiting. Collectively, these findings demonstrate that N3 is not needed to signal coincidently with or downstream of N1 during β-selection. Thus, ligand-activated N1 plays a unique role in this important developmental process. In addition, our data demonstrate that *N3* is not required for development of *ICN1*-induced T-LL. Development of T-LL in *Ikaros* mutant mice is characterized by somatic acquisition of activating *ICN1* mutations, but a recent study showed that T cell leukemogenesis in this model is also unaffected by *N3* deficiency [48]. Collectively, these data reveal that *N3* co-operation with *N1* is dispensable for both T cell development and T cell leukemogenesis.

In contrast to *N1* and *N2*, *N3* is not required for embryogenesis [49]. However, in post-natal mice *N3* critically regulates differentiation of vascular smooth muscle cells [50]. Indeed, *N3* mutations cause CADASIL, an autosomal dominant disease characterized by recurrent subcortical ischemic events and vascular dementia [51]. N3 also plays a role in other vascular pathologies [52]–[53] and has been implicated in some epithelial malignancies [54]–[55]. The clear involvement of N3 in a variety of human diseases makes it an attractive therapeutic target [54]–[56]. Our data suggest that selective inhibition of N3 activation for therapeutic purposes is unlikely to disrupt normal T cell development.

Why does *N3* loss-of-function have no impact on thymocyte β-selection and *ICN1*-induced T cell leukemogenesis? The fact that over-expression of ligand-independent *ICN3* can impact β-selection and drive T cell leukemogenesis [30] suggests that at least under these non-physiological circumstances, ICN3 can activate expression of ICN1 gene targets. However, our data suggest that N3 may not normally undergo ligand-dependent activation in the thymus. DL4, which is much more abundant than DL1 in the thymus, binds N1 strongly [29] and is non-redundantly required to activate N1 to drive T lineage commitment [6]–[7]. In contrast to the robust binding of DL4 by N1, we showed that N3 binds DL4 poorly. This finding suggests that N3 may not be efficiently activated by intrathymic DL4, although verification of this possibility awaits the development of reagents capable of detecting nuclear ICN3 in DN3 and pre-DP thymocytes.

Interestingly, reporter assays using promoters from different Notch target genes have demonstrated that N1 and N3 do not have identical transcriptional activities [57]. Furthermore, although N1 and N3 induce expression of many genes in common, N1 typically induces more robust expression [58]. Interestingly, ICN1 prefers closely spaced paired CSL binding sites, whereas ICN3 prefers single CSL sites with zinc finger transcription factor sites nearby [59]. ICN1 dimers co-operatively bind CSL paired sites [60], and importantly, ICN1 dimerization is required for both T cell development and leukemogenesis [61]. Thus, even if N3 is weakly activated by intrathymic DL4, it is not clear how efficiently physiologically expressed ICN3 would induce expression of critical *N1* target genes regulated by paired CSL sites, such as *Hes-1*, *Ptcra* and *c-myc*. Collectively, our finding that N3 binds DL4 poorly, combined with subtly different target site preferences for ICN1 vs ICN3 likely explain why N3 is dispensable for thymocyte β-selection, even when *N1* is limiting.
